# Assessment of Motor Dysfunction with Virtual Reality in Patients Undergoing [^123^I]FP-CIT SPECT/CT Brain Imaging

**DOI:** 10.3390/tomography7020009

**Published:** 2021-03-26

**Authors:** Jeanne P. Vu, Ghiam Yamin, Zabrina Reyes, Alex Shin, Alexander Young, Irene Litvan, Pengtao Xie, Sebastian Obrzut

**Affiliations:** 1 Department of Radiology, University of California San Diego School of Medicine, La Jolla, CA 92093, USA; jeanne.anh.vu@gmail.com (J.P.V.); gyamin@stanford.edu (G.Y.); zabrinareyes@gmail.com (Z.R.); alexyoung23j@gmail.com (A.Y.); 2 Department of Neuroimaging and Neurointervention, Stanford University Medical Center, Stanford, CA 94305, USA; 3 Department of Physics, Drexel University, Philadelphia, PA 19104, USA; itsalexshin@gmail.com; 4 Department of Neurology, University of California San Diego School of Medicine, La Jolla, CA 92093, USA; ilitvan@health.ucsd.edu; 5 Department of Electrical and Computer Engineering, University of California San Diego, La Jolla, CA 92093, USA; mrsebastian73@gmail.com

**Keywords:** virtual reality, Parkinson disease, motor dysfunction, SPECT, UPDRS

## Abstract

[^123^I]FP-CIT SPECT has been valuable for distinguishing Parkinson disease (PD) from essential tremor. However, its performance for quantitative assessment of motor dysfunction has not been established. A virtual reality (VR) application was developed and compared with [^123^I]FP-CIT SPECT/CT for detection of severity of motor dysfunction. Forty-four patients (21 males, 23 females, age 64.5 ± 12.4) with abnormal [^123^I]FP-CIT SPECT/CT underwent assessment of bradykinesia, activities of daily living, and tremor with VR. Support vector machines (SVM) machine learning models were applied to VR and SPECT data. Receiver operating characteristic (ROC) analysis demonstrated greater area under the curve (AUC) for VR (0.8418, 95% CI 0.6071–0.9617) compared with brain SPECT (0.5357, 95% CI 0.3373–0.7357, *p* = 0.029) for detection of motor dysfunction. Logistic regression identified VR as an independent predictor of motor dysfunction (Odds Ratio 326.4, SE 2.17, *p* = 0.008). SVM for prediction of the Unified Parkinson’s Disease Rating Scale Part III (UPDRS-III) demonstrated greater R-squared of 0.713 (*p* = 0.008) for VR, compared with 0.0764 (*p* = 0.361) for brain SPECT. This study demonstrates that VR can be safely used in patients prior to [^123^I]FP-CIT SPECT imaging and may improve prediction of motor dysfunction. This test has the potential to provide a simple, objective, quantitative analysis of motor symptoms in PD patients.

## 1. Introduction

Parkinson disease (PD) is a progressive disorder of the nervous system, resulting in the loss of dopaminergic neurons. PD patients require long-term treatment and frequent adjustments of symptomatic therapy as the disease progresses [1,2]. Dopamine transporter imaging with [^123^I]FP-CIT (*N*-(3-Fluoropropyl)-2β-carbomethoxy-3β-(4-[^123^I]iodophenyl) nortropane) single photon emission computed tomography (SPECT) and [^18^F]FP-CIT(*N*-(3-[^18^F]fluoropropyl)-2β-carboxymethoxy-3β-(4-iodophenyl)nortropane) positron emission tomography (PET) are in vivo molecular imaging techniques used to investigate loss of dopaminergic neurons in the striatum in patients suspected of having PD. Early diagnosis can improve the assessment of patient prognosis, as more than half of nigrostriatal dopaminergic neurons are lost before the appearance of typical motor manifestations [3]. [^123^I]FP-CIT SPECT is indicated in cases when the etiology of tremor and motor dysfunction is difficult to establish clinically [4]. Although this method can distinguish essential tremor from PD, a strong and consistent correlation between [^123^I]FP-CIT uptake in the striatum and disease severity, as assessed for example with the Movement Disorder Society-Sponsored Revision of the Unified Parkinson’s Disease Rating Scale (MDS-UPDRS) Part III (motor component), has not been established [5]. In a study by Beanamer et al., for example, the [^123^I]FP-CIT striatum to background activity ratios correlated with the bradykinesia subscores but not with rigidity or tremor subscores, suggesting that other factors, in addition to nigrostriatal degeneration, may contribute to motor dysfunction severity [6]. Simuni et al., reported a weak correlation between MDS-UPDRS-III and dopamine transporter binding in Parkinson’s Progression Markers Initiative (PPMI) cohort at the baseline with a Spearman correlation coefficient of −0.2119 for the contralateral putamen specific binding ratio [7]. Furthermore, [^18^F]FP-CIT PET demonstrated only modest correlations between radiotracer striatal uptake and UPDRS-III motor scores in 542 patients, with Pearson’s r of −0.128 and −0.155 for right and left posterior putamina, respectively [8]. Therefore, a novel, quantitative, facile, low-cost test that would complement dopamine transporter imaging and could be administered prior to radiotracer injection may be valuable for the objective assessment of motor dysfunction in early PD.

Virtual reality (VR) is an innovative technology that presents the user with interactive, realistic, computer-generated, and three-dimensional images [9]. VR allows for precise tracking of participant’s extremities and head, which may be helpful for quantitative assessment of movement abnormalities such as bradykinesia and tremor, both cardinal symptoms of PD. Furthermore, VR enables participants to perform tasks in custom, fully interactive environments that closely resemble activities of daily living, which become difficult for patients to perform as PD progresses. There is interest in the medical community to study VR in PD as a potential therapeutic tool. A pilot study demonstrated that movement imitation therapy with VR enhances the effect of motor practice in patients with PD [10]. VR dance exercise has been found to have a positive effect on balance, activities of daily living, and depressive disorder status of PD patients [11]. VR training significantly improved obstacle crossing performance and dynamic balance in participants with PD [12]. Canning et al., discussed the use of VR to study gait and balance through the manipulation of environments for improved understanding of motor-cognitive neural circuitry in PD [13]. According to a literature review by Mirelman et al., VR may be a promising tool for the assessment of gait impairments in PD [14]. Although VR approaches have been studied as a tool for therapy for PD patients, this proposal is, to our knowledge, the first systematic attempt to develop a VR methodology as a tool for quantifying motor dysfunction in patients undergoing dopamine transporter imaging with [^123^I]FP-CIT SPECT/CT.

## 2. Materials and Methods

### 2.1. Patients 

In this prospective study, fifty patients presenting for the evaluation of PD versus essential tremor with [^123^I]FP-CIT SPECT/CT or as part of enrollment in PPMI trial underwent testing with clinical questionnaires and VR between 6 September 2018 and 3 March 2020. Inclusion criteria were: age ≤85, Hoehn and Yahr scale ≤3, ability to provide oral and written informed consent, and ability to undergo VR testing in a seated position. Subjects were excluded if they had a normal brain [^123^I]FP-CIT SPECT/CT, a history of cerebral infarct, debilitating arthritis, or other orthopedic-relevant problem(s). Six patients were excluded on the basis of normal age-matched z-scores for putamen-to-background ratios on [^123^I]FP-CIT SPECT/CT, with the final group of patients enrolled in the study consisting of 44 patients. The study was approved by the Institutional Review Board.

### 2.2. Clinical Evaluations

Clinical evaluation included the assessment of the motor portion of the MDS Unified Parkinson’s Disease Rating Scale (UPDRS-III) as well as Hoehn and Yahr scale (H&Y) [15,16]. Motor evaluation was carried out with patients taking their usual antiparkinsonian medications for those patients with PD and already being treated. The presence of antiparkinsonian medications was recorded. Furthermore, participants underwent evaluation with Montreal Cognitive Assessment (MoCA) [17].

### 2.3. Virtual Reality

A room-scale VR environment with Vive (HTC Corporation, New Taipei City, Taiwan) headset and controllers was installed for the development of medical applications. The Vive device uses more than 70 sensors, including gyroscopes, accelerometers, and laser position sensors and can track the user’s head and arm movement with sub-millimeter precision. The Vive headset has the following specifications: resolution of 2160 × 1200 (1200 × 1080 per eye); physical size 19.0 × 12.7 × 8.9 cm, 563 g; sensors that include accelerometer and gyroscope; refresh rate of 90 Hz. The price of a Vive VR set is $400–$800 and includes two controllers, each with 24 sensors, a multi-function trackpad, dual-stage trigger, HD haptic feedback, integrated rechargeable 960 mAh battery, and weighing 1.125 lbs. VR application for quantification of motor dysfunction was developed in Unity software (Unity Technologies, San Francisco, CA, USA) version 2018.2.13f1 with 3D assets created in Autodesk Maya (Autodesk Inc., San Rafael, CA, USA).

**Figure 1:**
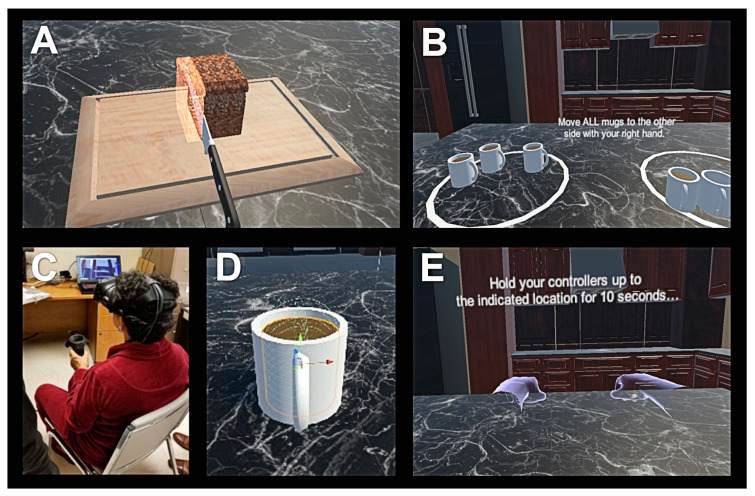
Screen outputs for virtual reality (VR) Time (**A**) and VR activities of daily living (ADL) Score (**B**) tests. Patient undergoing VR Time trial in a seated position (**C**). Close-up view of a mug containing liquid being designed for the VR ADL Score test (**D**). Screen output for the VR Posture Tremor test (**E**).

The VR test consisted of three modules that were completed in a seated position by the subject: (1) bradykinesia, (2) activities of daily living (ADL), and (3) tremor assessment. For (1), bradykinesia assessment, patients were asked to slice three food items (a loaf of bread, a carrot, and a cucumber) with two attempts for each hand using a 3D knife modeled in VR, as shown in Figure 1. The weight and size of the HTC Vive controller were similar to the weight and size of a typical kitchen knife. The amount of time patients needed to complete the food slicing activity was recorded for each hand (VR Time). For (2), ADL assessment, patients were prompted to pick up and move five mugs modeled in VR from one end of a virtual table to another, as shown in Figure 1. Each mug contained ten cylinders, which represented levels of hot liquid such as tea or coffee. If a particular mug were tilted in the vertical axis beyond a set threshold while being moved by the subject, the virtual liquid spilled out. The number of cylinders left in the mug after the patient placed it down on the table was recorded. The maximum score was 10 for each attempt (VR ADL Score). Five scores per hand were recorded for a total of ten attempts per subject. For (3), tremor assessment, an approach similar to a previously reported method that utilized a smartphone’s accelerometer in suspected PD patients was implemented [18]. First, subjects maintained both upper limbs fully extended in front of them with the palms facing the ground and holding the VR controllers for 10 s (‘Posture’ condition). Next, subjects sat quietly in a chair as relaxed as possible with forearms supported and with hands hanging and holding the VR controllers for 10 s (‘Rest’ condition). Two trials for each hand position were conducted while the 3D angular data from the controllers was recorded with a sampling rate of 90 Hz. Original and resampled data for each patient’s second trial with the left and right hand was used for further analysis with Matlab (MathWorks, Natick, MA, USA). Data acquired in the first 3 s was trimmed to mitigate for initial arm motion artifacts. Power spectral density was calculated by using the Welch periodogram with Matlab to obtain peak power of the controller position (m^2^/Hz) and dominant tremor frequency (Hz) [18,19]. 

### 2.4. Brain SPECT/CT Imaging

Patients were given two to three drops of Lugol’s solution orally and one hour later were injected with [^123^I]FP-CIT (111–185 MBq, 3–5 mCi, specific activity range, 2.5–4.5 × 10^14^ Bq/mmol). Following a four-hour uptake period, the patients underwent SPECT/CT imaging of the brain, utilizing a dual-head gamma camera (Optima NM/CT 640-GE Healthcare, Chicago, IL, USA) with a low-energy high-resolution collimator. After placing the patient’s head in a head holder, 120 views were acquired at 30 s each over 360 degrees. Images were reconstructed with Ordered Subsets Expectation Maximization algorithm, using a 128 × 128 matrix. Attenuation correction was applied using CT image data and Chang’s method. Reconstructed brain SPECT images were registered to a template based on a database of healthy controls (DaTQUANT software, GE Healthcare, Chicago, IL, USA) to perform semiquantitative analysis of nigrostriatal degeneration [20]. Automatically generated volumes of interest were used to obtain striatal uptake parameters, including putamen-to-caudate ratio and Specific Binding Ratios (SBRs) for striatum-to-background, caudate-to-background, putamen-to-background, and with SBR calculated as (target region/reference region) −1 [21]. Corresponding age-matched z-scores were calculated using the following formula: z-score  =  (individual SBR − mean SBR in normal database)/standard deviation of SBR in normal database. 

### 2.5. Statistical Analysis

Differences between sample means based on laterality for VR and [^123^I]FP-CIT SPECT DaTQUANT results were compared using Student’s *t*-test. Patients with a clinically important difference (CID) in motor dysfunction were identified based on the UPDRS-III motor score >10 points threshold. This CID estimate has previously been determined to be potentially clinically meaningful for detecting changes in PD progression and response to therapeutic interventions [22]. Variables generated with VR testing and brain SPECT striatal binding parameters combined with the presence or absence of PD medications were entered into a support vector machines (SVM) machine learning classification model for detection of CID in motor dysfunction using linear kernel. Analysis was performed with and without 5-fold cross validation with Matlab. SVM scores consisting of posterior probabilities were used to generate receiver operating characteristic curves (ROC). The ROC areas under the curve (AUCs) were compared with ROCKIT software (Metz ROC Software, University of Chicago, Chicago, IL, USA) [23,24]. Multiple features, including age, male gender, right hand dominance, and presence of PD medications as well as VR ADL Scores and SPECT scores from SVM were entered into a logistic regression model. Statistically significant contribution of each feature for the detection of CID in motor dysfunction was evaluated with Matlab. Variables generated with VR testing and brain SPECT striatal binding parameters combined with the presence or absence of PD medications were entered into a SVM machine learning regression model for prediction of UPDRS-III motor scores. Patients with missing values were excluded and analysis was performed with and without hold-out validation with Matlab, using 70% of data for training and 30% for testing. Mean squared errors (MSE), R-squared coefficients, and *p*-values were calculated to evaluate the goodness of fit of the SVM regression models. 

## 3. Results

Table 1 contains demographics as well as results of UPDRS-III and MoCA questionnaires. Mean patient age was 64.5 ± 12.4 and there were more women than men enrolled in the study. Twenty-five out of forty-four patients were classified with CID in motor dysfunction. Twelve patients were receiving medication for PD in the study, including levodopa, dopamine agonists, monoamine oxidase type B (MAO-B) inhibitors, or amantadine. While a greater number of patients were right-handed than left-handed, there was no difference in mean VR results or mean [^123^I]FP-CIT SPECT results based on laterality, except for a greater mean VR ADL Score for the left upper extremity (9.2 ± 0.9) compared with right upper extremity (8.5 ± 1.2, *p* = 0.003), as shown in Table 2. 

**Figure 2:**
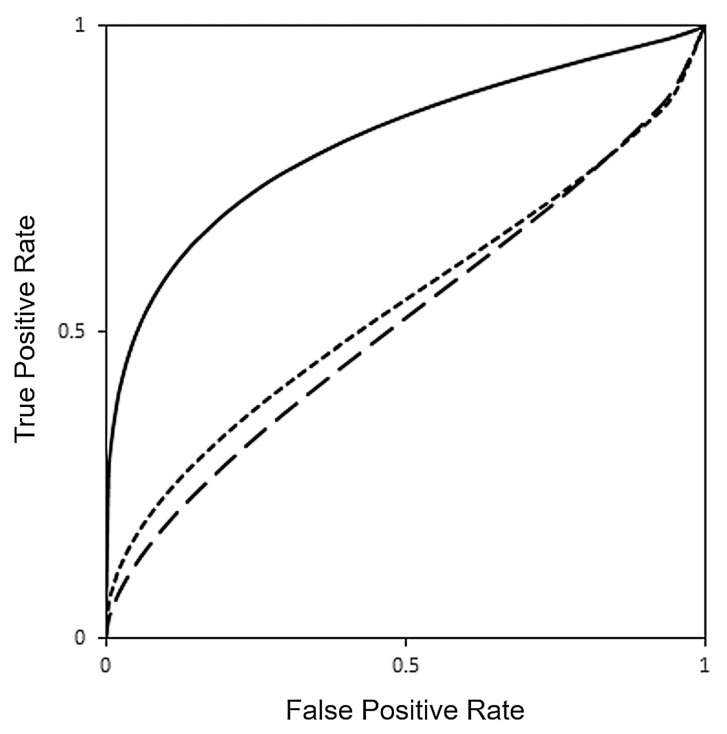
Receiver operating characteristic (ROC) curves for detection of clinically important difference (CID) in motor dysfunction with VR (continuous line, AUC = 0.8418), [^123^I]FP-CIT SPECT (long dashed line, AUC = 0.5357) and [^123^I]FP-CIT SPECT with PD medications (short dashed line, AUC = 0.5397) obtained using a SVM machine learning classification model.

SVM classification algorithm was trained to detect CID in motor dysfunction with VR and SPECT data. ROC curve analysis demonstrated greater AUC for VR (AUC = 0.8418) compared with brain [^123^I]FP-CIT SPECT imaging (AUC = 0.5357, *p* = 0.029). Furthermore, AUC for SPECT improved only minimally (AUC = 0.5397) when the presence of PD medication was included with training data but still remained lower than AUC for VR in detection of motor dysfunction (*p* = 0.042), as shown in Table 3 and Figure 2. Logistic regression identified the cross-validated VR SVM score as the only predictive feature for detection of CID in motor dysfunction (Odds Ratio 326.4, SE 2.17, *p* = 0.008), compared with age, gender, hand dominance, presence of PD medication, and cross-validated [^123^I]FP-CIT SPECT SVM score (Table 4). 

**Figure 3:**
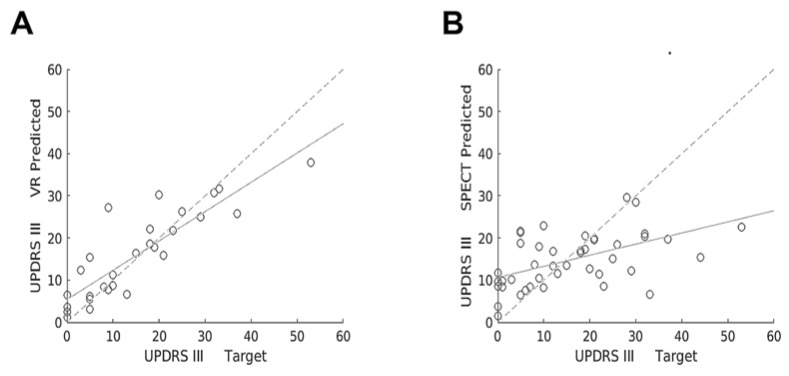
Scatter plots showing target versus predicted UPDRS-III scores with VR (**A**) and [^123^I]FP-CIT SPECT (**B**) obtained using a regression SVM machine learning model for the entire data set. Linear regression lines show correlation between target versus predicted UPDRS-III scores (continuous lines) and target versus target scores (dashed lines).

SVM regression algorithm was trained to predict UPDRS-III motor score with VR and SPECT data. Hold-out validation testing demonstrated greater R-squared for VR (R-squared = 0.713, *p* = 0.008), compared with brain [^123^I]FP-CIT SPECT imaging (R-squared = 0.0764, *p* = 0.361). Figure 3 shows scatter plots comparing target versus predicted UPDRS-III scores with VR and [^123^I]FP-CIT SPECT, obtained using a regression SVM model for the entire data set. Furthermore, R-squared for SPECT did not improve significantly (R-squared = 0.0676, *p* = 0.391) when the presence of PD medication was included with training data, as depicted in Table 5.

**Figure 4:**
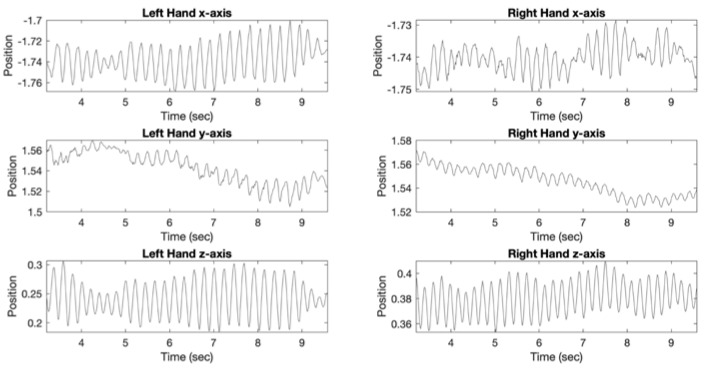
Plots demonstrating position (*x*, *y*, and *z* axis) versus time for Posture VR tremor assessment in a 67-year-old right-handed female. Analysis of left upper extremity VR data demonstrated VR Posture Tremor Power of 7.26 × 10^−4^ m^2^/Hz and VR Posture Tremor Frequency of 4.7 Hz. Analysis of right upper extremity VR data demonstrated VR Posture Tremor Power of 1.26 × 10^−4^ m^2^/Hz and VR Posture Tremor Frequency of 5.4 Hz. MoCA score was 21 and UPDRS-III motor score was 21 with tremor sub-scores (UPDRS 3.15, 3.17, 3.18) ranging from 1 (slight) to 2 (moderate). Patient was not receiving medication for PD.

Figure 4 shows plots demonstrating hand position versus time for Posture tremor assessment with VR in a 67-year-old right-handed female. This patient only received non-PD medications, including alendrolate, gabapentin, and mirabegron. MoCA score was 21 and UPDRS-III motor score was 21. UPDRS-III item 3.18 bilateral tremor sub-score for this subject was 1, indicating slight rest tremor constancy. Left hand UPDRS-III tremor sub-scores in this patient were: item 3.15b-2, mild postural tremor; item 3.17b-1 slight rest tremor amplitude. Analysis of left upper extremity VR data in this subject yielded VR Posture Tremor Power of 7.26 × 10^−4^ m^2^/Hz and VR Posture Tremor Frequency of 4.7 Hz. For comparison, Table 2 demonstrated mean Left VR Posture Tremor Power of 2.13 × 10^−5^ ± 1.23 × 10^−4^ m^2^/Hz and mean Left VR Posture Tremor Frequency of 2.3 ± 1.8 Hz. Right hand UPDRS-III tremor sub-scores in this patient were: item 3.15a-2, mild postural tremor; item 3.17a-2, mild rest tremor amplitude. Analysis of right upper extremity VR data in this subject yielded VR Posture Tremor Power of 1.26 × 10^−4^ m^2^/Hz and VR Posture Tremor Frequency of 5.4 Hz. For comparison, Table 2 demonstrated mean Right VR Posture Tremor Power of 4.86 × 10^−6^ ± 2.18 × 10^−5^ m^2^/Hz and mean Right VR Posture Tremor Frequency of 2.1 ± 1.3 Hz. Therefore, VR results matched with UPDRS-III total and sub-score results for tremor quantification in this patient.

**Figure 5:**
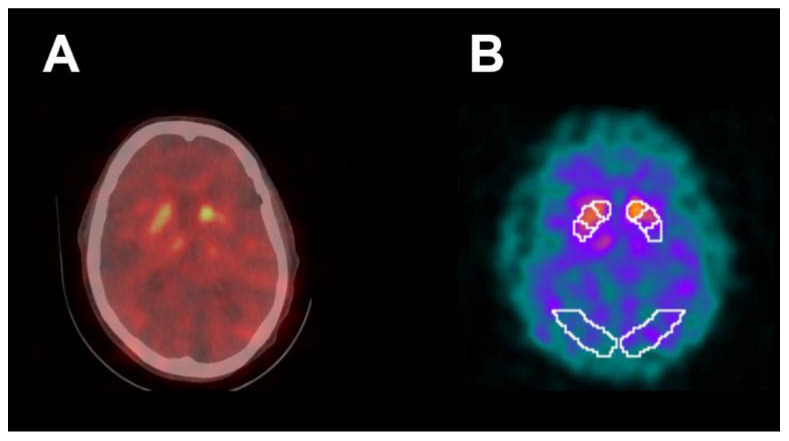
Abnormal [^123^I]FP-CIT SPECT/CT (**A**) in a 76-year-old right-handed male demonstrating decreased activity in the striatum bilaterally, particularly in the left putamen. [^123^I]FP-CIT SPECT images (**B**) co-registered to the DaTQUANT software template demonstrated a left putamen SBR of 0.6 with z-score of −3.93. UPDRS-III motor score was 5 and MoCA score was 20. Patient was not receiving medication for PD. For contralateral right upper extremity, VR time was 50 s, VR ADL Score was 9.8, VR Rest Tremor Power was 1.48 × 10^−8^ m^2^/Hz, and VR Posture Tremor Power was 6.09 × 10^−8^ m^2^/Hz.

Figure 5 shows abnormal [^123^I]FP-CIT SPECT/CT in a 76-year-old right-handed male indicating decreased activity in the striatum bilaterally, particularly in the left putamen (SBR z-score −3.93). MoCA score was 20 and UPDRS-III motor score was 5. For comparison, mean UPDRS-III was 16.3 ± 13.0 (Table 1) in all study subjects. This patient was not receiving medication for PD or any other illness. For the contralateral right upper extremity, VR Time was 50 s, VR ADL Score was 9.8, VR Rest Tremor Power was 1.48 × 10^−8^ m^2^/Hz and VR Posture Tremor Power was 6.09 × 10^−8^ m^2^/Hz. For comparison, in all study subjects the right upper extremity mean VR Time was 72.1 ± 47.6 s, mean VR ADL Score was 8.5 ± 1.2, mean VR Rest Tremor Power was 6.73 × 10^−6^ ± 2.99 × 10^−5^ m^2^/Hz, and VR Posture Tremor Power was 4.86 × 10^−6^ ± 2.18 × 10^−5^ m^2^/Hz (Table 2). Therefore, VR results matched with UPDRS-III motor evaluation in this patient, while [^123^I]FP-CIT SPECT/CT results were discordant with UPDRS-III.

## 4. Discussion

This study demonstrates that VR is a novel software and hardware tool that can be safely used for the improved assessment of motor dysfunction in patients undergoing [^123^I]FP-CIT SPECT/CT brain imaging. Although SPECT has been very helpful in distinguishing essential tremor from early PD in the clinical setting, a robust association between [^123^I]FP-CIT uptake in the striatum and severity of motor dysfunction has not been confirmed [5,6,8]. Similarly, our study did not identify a strong correlation between [^123^I]FP-CIT SPECT/CT semi-quantitative measurements and UPDRS-III motor scores despite using a SVM machine learning algorithm to improve performance. Figure 5, for example, shows a markedly decreased putamen SBR on brain SPECT/CT in a 76-year-old right-handed male patient who was not receiving medication for PD with a discordant, borderline-normal UPDRS-III motor score. Furthermore, our results showed a small AUC under the ROC curve for detection of clinically significant motor dysfunction with [^123^I]FP-CIT SPECT/CT, suggesting both low sensitivity and specificity. 

Since patients undergoing [^123^I]FP-CIT SPECT/CT imaging spend one hour waiting for injection of radiotracer after administration of oral iodine drops according to the established protocol, this presents an opportunity to administer a non-invasive test in an attempt to improve the assessment of motor dysfunction. We performed the VR testing in this time window that included the evaluation of bradykinesia, ADL, and tremor. The patients reported positive feedback as well as high motivation for completing VR testing and no significant side effects. VR yielded greater AUC under the ROC curve for detection of clinically significant motor dysfunction than [^123^I]FP-CIT SPECT/CT. Furthermore, VR showed improved the correlation for prediction of the UPDRS-III score compared with SPECT. Figure 4 shows VR tremor test results that matched with UPDRS-III tremor sub-scores in a 67-year-old right-handed female patient. Since this is the first study using VR to measure motor dysfunction in a patient population undergoing [^123^I]FP-CIT SPECT/CT brain imaging, there is potential for further improving VR performance with test optimization and implementation of more advanced technologies such as augmented reality, a smaller headset, and glove controllers that can track the motion of the upper extremity digits [25].

Although the VR software applications used in this study may have the potential to provide a quantitative analysis of motor symptoms, it has limitations in assessment of PD patients, due to its current inability to evaluate non-motor symptoms [26]. In addition to motor symptoms such as tremor, slowness of movement, and stiffness, most people develop other health problems related to PD. These non-motor symptoms (NMS) include depression, psychosis, constipation, erectile dysfunction, hypotension, and sleep disorders [27]. NMS are common and can be more troublesome and disabling than motor symptoms. Despite their major impact on quality of life and the fact that they can often be easily treated by primary care practitioners and neurologists with medications and other novel strategies, NMS are usually under-recognized and untreated in clinical practice. In light of the importance and the impact that NMS hold for persons with PD and for their families, it is vitally important that physicians caring for them are knowledgeable and skilled at diagnosing NMS. This task can be challenging because patients themselves may not mention these symptoms to their physicians, either because they do not associate them with PD or, in some instances, out of embarrassment. Therefore, screening tools have been developed for use in research and clinical practice, including PD specific questionnaires (such as the Non-Motor Symptoms Questionnaire [NMS-Quest] published in 2006) to improve detection of NMS. Therefore, future development of VR could include questionnaire-based items that ask the patient about non-motor symptoms. Recently, Lim JE et al., developed a novel, fully immersive VR system (CAVIRE: Cognitive Assessment by VIrtual REality), which incorporates automated audio-visual instructions to assess the six domains of cognition [28]. A similar approach could be explored with VR for the evaluation of NMS to provide a more comprehensive automated testing of PD severity and progression.

One of the limitations of this study was the small sample size. Furthermore, a smaller number of patients underwent complete VR testing, compared with the number of patients undergoing brain imaging with SPECT/CT. This was due to the variable amount of time it took to administer clinical surveys (UPDRS-III, MoCA) in different patients, subsequently limiting time left for VR testing in some patients prior to radiotracer injection. Due to time constraints between administration of Lugol’s solution and radiotracer injection, only a limited cognitive assessment was performed with MoCA. A more comprehensive future study of the potential influence of cognitive impairment on VR testing could also include administering the mini-mental state examination (MMSE) and Clinical Dementia Rating scale Sum of Boxes (CDR-SB). To account for issues related to patient understanding of the VR equipment, patients were allowed two attempts to complete the assigned tasks. Nevertheless, we did not assess the potential range of variability in VR test performance in each patient throughout the day (i.e., assessing VR tasks before and after the brain imaging), which could be affected by the level of fatigue, alertness, and overall motivation to perform the task. The VR tasks represent a subset of measures that are assessed in UPDRS-III. UPDRS-III includes several measures that were not assessed by our VR test, such as speech, facial expression, posture, gait, and postural stability. Some of these measures were not included in the VR test simply due to safety concerns in patients with a potential movement disorder standing or walking. Therefore, the tasks were performed while sitting, which can be seen as a limited corollary of the UPDRS-III. Since the prediction of H&Y stage was not performed with VR, future studies can explore prediction of both UPDRS-III and H&Y stage. However, predicting both parameters simultaneously may require a more complex machine learning algorithm than SVM, such as deep learning. Statistical analysis examined the effect of levodopa and all other PD medications collectively (Table 4) and did not evaluate the specific effect of levodopa alone on the results. There was a disproportionate number of right-hand dominant subjects enrolled in this study (86%, 38/44). Nevertheless, more than an expected number of left-hand dominant subjects (14%, 6/44)) enrolled in the study, given that 10% of the world’s population is left-handed. This difference may partially explain better VR ADL Scores in subjects enrolled using their left hand to perform the ADL task compared with the right hand, as shown in Table 2. It is unclear if the size and weight of the controllers and tracking device may have an influence in how long it takes for patients to complete tasks. Since this is an exploratory study to demonstrate the feasibility of VR for evaluation of PD motor dysfunction, future studies may employ controllers and tracking devices that are becoming smaller and lighter.

## 5. Conclusions

This study demonstrated that a novel computer-generated VR environment may safely and effectively provide a simple, rapid, quantitative assessment of motor dysfunction in patients being evaluated for the presence of PD and may serve as an adjunct to brain imaging such as [^123^I]FP-CIT SPECT/CT. Future studies will assess the utility of serial VR testing in a larger number of patients to evaluate motor dysfunction progression in PD and its potential to impact clinical management.

## Figures and Tables

**Table 1:** Demographics and questionnaire results for patients enrolled in the study.

	N (%) or Mean ± Standard Deviation (Range)
Male	21/44 (48%)
Female	23/44 (52%)
Age	64.5 ± 12.4 (36–85)
UPDRS-III	16.3 ± 13.0 (0–53)
UPDRS-III > 10	25/44 (57%)
PD Meds	12/44 (27%)
H&Y	1 ± 0.9 (0–3)
MoCA	22.6 ± 6.1 (9–30)

Abbreviations: UPDRS, Unified Parkinson’s Disease Rating Scale; PD Meds, patients receiving medical therapy for Parkinson disease (PD); H&Y, Hoehn and Yahr scale; MoCA, Montreal Cognitive Assessment.

**Table 2:** Hand dominance, virtual reality (VR), and [^123^I]FP-CIT SPECT results based on laterality.

	Right	Left	*p*-Value
Hand Dominance	38	6	NA
SPECT Striatum SBR	1.6 ± 0.7	1.6 ± 0.7	1.000
SPECT Caudate SBR	1.9 ± 0.8	1.9 ± 0.7	1.000
SPECT Putamen SBR	1.5 ± 0.7	1.5 ± 0.7	1.000
SPECT Putamen-To-Caudate Ratio	0.9 ± 0.1	0.9 ± 0.1	1.000
VR Time (s)	72.1 ± 47.6	83.7 ± 60.1	0.318
VR ADL Score	8.5 ± 1.2	9.2 ± 0.9	0.003 *
VR Posture Tremor Frequency (Hz)	2.1 ± 1.3	2.3 ± 1.8	0.552
VR Posture Tremor Power (m^2^/Hz)	4.86 × 10^−6^ ± 2.18 × 10^−5^	2.13 × 10^−5^ ± 1.23 × 10^−4^	0.385
VR Rest Tremor Frequency (Hz)	3.3 ± 2.0	3.4 ± 2.1	0.552
VR Rest Tremor Power (m^2^/Hz)	6.73 × 10^−6^ ± 2.99 × 10^−5^	1.38 × 10^−6^ ± 7.41 × 10^−6^	0.253

Abbreviation: SBR, Specific Binding Ratio; * significant (*p*-value < 0.05).

**Table 3:** Receiver operating characteristic (ROC) AUCs for detection of clinically important difference (CID) in motor dysfunction with VR, [^123^I]FP-CIT SPECT, and SPECT + PD Meds using SVM.

	VR #	VR	SPECT	SPECT + PD Meds
AUC	0.9133	0.8418	0.5357	0.5397
AUC 95% CI	(0.6350–1.0000)	(0.6071–0.9617)	(0.3373–0.7357)	(0.3374–0.7345)
SE	0.0577	0.0770	0.1038	0.1037
*p*-value vs. VR AUC	NA	NA	0.029 *	0.042 *

Abbreviations: AUC, area under the curve; SVM, support vector machines; # without k-fold cross validation; CI, confidence interval; SE, standard error; PD Meds-feature accounting for patients receiving medical therapy for Parkinson disease (PD); * significant (*p*-value < 0.05).

**Table 4:** Logistic regression results comparing age, gender, hand dominance, Parkinson disease (PD) medications, as well as VR and [^123^I]FP-CIT SPECT SVM scores for detection of CID in motor dysfunction.

	Beta	SE	OR	*p*-Value
Age	−0.01402	0.05022	0.9861	0.780
Male Gender	−1.1185	1.2214	0.3268	0.360
Right Hand Dominance	1.2458	1.226	3.4756	0.319
PD Meds	−0.13796	0.16178	0.8711	0.394
VR SVM Score	5.7881	2.172	326.4029	0.008 *
SPECT SVM Score	7.353	53.781	1560.9	0.891

Abbreviations: VR, virtual reality; SVM, support vector machines; Beta, logistic regression coefficients; SE, Standard Error; OR, odds ratio; PD Meds-feature accounting for patients receiving medical therapy for Parkinson disease (PD); * significant (*p*-value < 0.05).

**Table 5:** MSE and R-squared coefficients for VR, [^123^I]FP-CIT SPECT, and SPECT + PD Meds in prediction of UPDRS-III motor score with SVM.

	VR			SPECT			SPECT + PD Meds		
MSE	41.6915			122.6062			122.4380		
	N	R-Squared	*p*-Value	N	R-Squared	*p*-Value	N	R-Squared	*p*-Value
All	28	0.755	0.001 *	44	0.272	0.001 *	44	0.273	0.001 *
Train	20	0.729	0.001 *	41	0.254	0.004 *	41	0.273	0.004 *
Test	8	0.713	0.008 *	13	0.0764	0.361	13	0.0676	0.391

Abbreviations: MSE, resubstitution (in-sample) mean-squared error; VR, virtual reality; PD Meds-feature accounting for patients receiving medical therapy for Parkinson disease (PD); * significant (*p*-value < 0.05).

## Data Availability

The data presented in this study are available on request from the corresponding author. The data are not publicly available to preserve patient confidentiality.

## References

[1] Rizek P., Kumar N., Jog M.S. (2016). An update on the diagnosis and treatment of Parkinson disease. Cmaj.

[2] Visanji N.P., Brotchie J.M., Kalia L.V., Koprich J.B., Tandon A., Watts J.C., Lang A.E. (2016). α-Synuclein-Based Animal Models of Parkinson’s Disease: Challenges and Opportunities in a New Era. Trends Neurosci..

[3] Bernheimer H., Birkmayer W., Hornykiewicz O., Jellinger K., Seitelberger F. (1973). Brain dopamine and the syndromes of Parkinson and Huntington Clinical, morphological and neurochemical correlations. J. Neurol. Sci..

[4] Djang D.S.W., Janssen M.J.R., Bohnen N., Booij J., Henderson T.A., Herholz K., Minoshima S., Rowe C.C., Sabri O., Seibyl J. (2012). SNM practice guideline for dopamine transporter imaging with 123I-ioflupane SPECT 1.0. J. Nucl. Med..

[5] Ba F., Martin W.R.W. (2015). Dopamine transporter imaging as a diagnostic tool for parkinsonism and related disorders in clinical practice. Park. Relat. Disord..

[6] Benamer H.T.S., Patterson J., Wyper D.J., Hadley D.M., Macphee G.J.A., Grosset D.G. (2000). Correlation of Parkinson’s disease severity and duration with 123I-FP-CIT SPECT striatal uptake. Mov. Disord..

[7] Simuni T., Siderowf A., Lasch S., Coffey C.S., Caspell-Garcia C., Jennings D., Tanner C.M., Trojanowski J.Q., Shaw L.M., Seibyl J. (2018). Longitudinal Change of Clinical and Biological Measures in Early Parkinson’s Disease: Parkinson’s Progression Markers Initiative Cohort. Mov. Disord..

[8] Yang Y., Cheon M., Kwak Y.T. (2017). 18F-FP-CIT Positron Emission Tomography for Correlating Motor and Cognitive Symptoms of Parkinson’s Disease. Dement. Neurocogn. Disord..

[9] Riva G., Baños R.M., Botella C., Mantovani F., Gaggioli A. (2016). Transforming experience: The potential of augmented reality and virtual reality for enhancing personal and clinical change. Front. Psychiatry.

[10] Robles-García V., Corral-Bergantiños Y., Espinosa N., García-Sancho C., Sanmartín G., Flores J., Cudeiro J., Arias P. (2016). Effects of movement imitation training in Parkinson’s disease: A virtual reality pilot study. Park. Relat. Disord..

[11] Lee N.Y., Lee D.K., Song H.S. (2015). Effect of virtual reality dance exercise on the balance, activities of daily living, And depressive disorder status of Parkinson’s disease patients. J. Phys. Ther. Sci..

[12] Liao Y.Y., Yang Y.R., Cheng S.J., Wu Y.R., Fuh J.L., Wang R.Y. (2015). Virtual Reality-Based Training to Improve Obstacle-Crossing Performance and Dynamic Balance in Patients with Parkinson’s Disease. Neurorehabil. Neural Repair.

[13] Canning C.G., Allen N.E., Nackaerts E., Paul S.S., Nieuwboer A., Gilat M. (2020). Virtual reality in research and rehabilitation of gait and balance in Parkinson disease. Nat. Rev. Neurol..

[14] Mirelman A., Maidan I., Deutsch J.E. (2013). Virtual reality and motor imagery: Promising tools for assessment and therapy in Parkinson’s disease. Mov. Disord..

[15] Goetz C.G., Fahn S., Martinez-Martin P., Poewe W., Sampaio C., Stebbins G.T., Stern M.B., Tilley B.C., Dodel R., Dubois B. (2007). Movement disorder society-sponsored revision of the unified Parkinson’s disease rating scale (MDS-UPDRS): Process, format, and clinimetric testing plan. Mov. Disord..

[16] Hoehn M.M., Yahr M.D. (1967). Parkinsonism: Onset, progression, and mortality. Neurology.

[17] Nasreddine Z.S., Phillips N.A., Bédirian V., Charbonneau S., Whitehead V., Collin I., Cummings J.L., Chertkow H. (2005). The Montreal Cognitive Assessment, MoCA: A brief screening tool for mild cognitive impairment. J. Am. Geriatr. Soc..

[18] Barrantes S., Sánchez Egea A.J., González Rojas H.A., Martí M.J., Compta Y., Valldeoriola F., Mezquita E.S., Tolosa E., Valls-Solè J. (2017). Differential diagnosis between Parkinson’s disease and essential tremor using the smartphone’s accelerometer. PLoS ONE.

[19] Halliday D.M., Rosenberg J.R., Amjad A.M., Breeze P., Conway B.A., Farmer S.F. (1995). A framework for the analysis of mixed time series/point process data-Theory and application to the study of physiological tremor, single motor unit discharges and electromyograms. Prog. Biophys. Mol. Biol..

[20] Varrone A., Dickson J.C., Tossici-Bolt L., Sera T., Asenbaum S., Booij J., Kapucu O.L., Kluge A., Knudsen G.M., Koulibaly P.M. (2013). European multicentre database of healthy controls for [123I]FP- CIT SPECT (ENC-DAT): Age-related effects, gender differences and evaluation of different methods of analysis. Eur. J. Nucl. Med. Mol. Imaging.

[21] Marek K., Chowdhury S., Siderowf A., Lasch S., Coffey C.S., Caspell-Garcia C., Simuni T., Jennings D., Tanner C.M., Trojanowski J.Q. (2018). The Parkinson’s progression markers initiative (PPMI)–establishing a PD biomarker cohort. Ann. Clin. Transl. Neurol..

[22] Shulman L.M., Gruber-Baldini A.L., Anderson K.E., Fishman P.S., Reich S.G., Weiner W.J. (2010). The clinically important difference on the unified parkinson’s disease rating scale. Arch. Neurol..

[23] Metz C.E., Herman B.A., Shen J.H. (1998). Maximum likelihood estimation of receiver operating characteristic (ROC) curves from continuously-distributed data. Stat. Med..

[24] Metz C.E., Herman B.A., Roe C.A. (1998). Statistical comparison of two ROC-curve estimates obtained from partially-paired datasets. Med. Decis. Mak..

[25] Lee A., Hellmers N., Vo M., Wang F., Popa P., Barkan S., Patel D., Campbell C., Henchcliffe C., Sarva H. (2020). Can google glass^TM^ technology improve freezing of gait in parkinsonism? A pilot study. Disabil. Rehabil. Assist. Technol..

[26] Pfeiffer R.F. (2016). Non-motor symptoms in Parkinson’s disease. Park. Relat. Disord..

[27] Baig F., Lawton M., Rolinski M., Ruffmann C., Nithi K., Evetts S.G., Fernandes H.R., Ben-Shlomo Y., Hu M.T.M. (2015). Delineating nonmotor symptoms in early Parkinson’s disease and first-degree relatives. Mov. Disord..

[28] Lim J.E., Wong W.T., Teh T.A., Lim S.H., Allen J.C., Quah J.H.M., Malhotra R., Tan N.C. (2021). A Fully-Immersive and Automated Virtual Reality System to Assess the Six Domains of Cognition: Protocol for a Feasibility Study. Front. Aging Neurosci..

